# First Comprehensive Global Bibliometric Analysis of Monkeypox Virus Research Landscape From 1976 to 2025

**DOI:** 10.1155/cjid/2362823

**Published:** 2026-05-24

**Authors:** Uwem Okon Edet, Md Zulfekar Ali, Bassey Ini Ubi, Edema Enogiomwan Imalele, Bassey Edet, Francisca Nwaokorie, Clement Meseko

**Affiliations:** ^1^ Infectious & Transboundary Animal Diseases, National Veterinary Research Institute (NVRI), Vom, 930101, Nigeria, nvri.gov.ng; ^2^ Department of Biological Science (Microbiology Unit), Arthur Jarvis University, Akpabuyo, Nigeria; ^3^ Department of Microbiology, School of Pure and Applied Sciences, Federal University of Technology, Ikot Abasi, Akwa Ibom, Nigeria, futa.edu.ng; ^4^ Animal Health Research Division, Bangladesh Livestock Research Institute, Savar, Dhaka, 1341, Bangladesh, blri.gov.bd; ^5^ Department of Microbiology, Faculty of Biological Sciences, University of Calabar, Calabar, Cross River, Nigeria, unical.edu.ng; ^6^ Department of Zoology and Environmental Biology, Faculty of Biological Sciences, University of Calabar, Calabar, PMB1115, Cross River, Nigeria, unical.edu.ng; ^7^ Department of Medical Laboratory Science, College of Medicine, University of Lagos, Lagos, Nigeria, unilag.edu.ng

**Keywords:** bibliometric analysis, emerging infectious diseases, global collaboration, monkeypox virus, research trends

## Abstract

**Background:**

Six decades have elapsed since the index case of monkeypox virus (MPXV) was reported. Unlike most DNA viruses characterized by slow mutation and spread, MPXV caused the global outbreak of 2022, and as expected, several studies prior and post the 2022 outbreak have emanated. Despite its ability to trigger a potential pandemic, there is no holistic quantitative and qualitative insight into MPXV research landscape globally.

**Methods:**

Following the Scientific Procedures and Rationales for Systematic Literature Review (SPAR‐4‐SLR protocol) applied to the Scopus database, studies were retrieved covering 1976–2025 on MPXV globally and screened using predefined criteria. The final dataset was analyzed using Biblioshiny, RStudio, and VOSviewer tool.

**Results:**

Of the 8460 retrieved records, 5315 met the inclusion and exclusion criteria, comprising 79.98% original articles and 20.02% review articles, authored by 32,098 researchers across 1547 journals. Our analysis of the MPXV research revealed an average annual growth rate of 12.38%, with a pronounced surge after 2021, coinciding with the global 2022 outbreak. The United States dominated research output, authorship, funding, institutional leadership, and international collaborations, with the Centers for Disease Control (CDC) emerging as the most influential institution. China, India, and the United Kingdom also contributed substantially, while resource‐poor settings were underrepresented. Keyword and thematic analyses revealed a transition from early virological and animal studies to recent emphasis on human transmission, epidemiology, vaccination, and therapeutics. Highly cited studies were concentrated in leading infectious disease journals. Overall, MPXV research has evolved rapidly, highlighting expanding global collaboration and shifting research priorities.

**Conclusion:**

The study demonstrates a sharp post‐2021 expansion of MPXV research, dominated by high‐income countries, particularly the United States. Strengthening research contributions, surveillance, and collaborations from MPXV‐endemic regions in Africa is essential for balanced global preparedness and response efforts.

## 1. Introduction

The Monkeypox virus (MPXV), a member of the genus *Orthopoxvirus*, was first detected in 1959 after a series of eight cases between 1958 and 1968 [1]. The first recognized human case occurred in 1970 in the Democratic Republic of the Congo (DRC), involving a nine‐month‐old infant [2]. Since then, sporadic infections and localized outbreaks have continued across Central and West Africa [3]. Global attention toward MPXV increased in 2003 when it was first detected outside Africa, in the United States of America (USA) [4]. The 2022 global outbreak led the World Health Organization (WHO) to declare MPXV as a virus of Public Health Emergency of International Concern, as it affected more than 120 countries, from which over 100,000 laboratory‐confirmed infections and more than 220 deaths were recorded [5]. As noted by Islam et al. [6], the 2022 global outbreak represents the largest global spread of the virus, extending beyond its traditional endemic regions in Central and West Africa to several nonendemic countries. The origin of the 2022 global outbreak is suspected to have originated in Nigeria [7]. A five‐decade systematic review estimated the overall MPXV case fatality rate to be approximately 4%, with higher mortality reported in earlier outbreaks and among children, while recent outbreaks show declining fatality due to improved surveillance and clinical management [8].

MPXV is a zoonotic pathogen whose putative reservoirs include rodents, monkeys, apes, and humans [9]. Among these hosts, humans are the susceptible and most affected. Transmission occurs through close human contacts, via contaminated materials, and zoonotic exposures, with waning smallpox immunity and increased international travel contributing to rapid dissemination [6]. Specifically, transmission routes include contact with mucous membranes or broken skin, direct physical contact, sexual activity, and exposure to contaminated needles and materials such as bedding and clothing [10, 11]. Additional infection routes include inhalation of respiratory droplets, handling infected animal fluids, and contact during bushmeat processing and hunting [8, 10–12]. An earlier study aimed at evaluating the association between cases following the global outbreak of 2022, and meteorological factors showed that temperature, surface pressure, and relative humidity had positive impact on cases or incidence, while dew/frost point, precipitation, and wind speed had a negative significant impact [13].

The incubation period typically ranges from 4 to 21 days, averaging 6–13 days [14, 15]. Infected individuals remain contagious from symptom onset until lesions have crusted and fallen off and new skin has formed [16, 17]. Clinical manifestations begin with fever, myalgia, and sore throat, progressing to a painful or itchy rash, lymphadenopathy, headache, and fatigue [18]. Other reported symptoms include oropharyngeal lesions, conjunctivitis, blepharitis, cough, and cervical or generalized lymph node enlargement [1]. As expected, numerous studies have been published over the last six and a half decades examining various aspects of MPXV.

To understand the research landscape, several bibliometric studies on MPXV have emerged and these studies have only focused on certain aspects of the virus. Khaleel et al. [19] concentrated specifically on the application of artificial intelligence to MPXV, mapping trends in machine learning–based diagnosis and forecasting, yet their analysis did not cover the broader epidemiological, clinical, or genomic landscape of MPXV research. Ravichandran and Rajendran’s scientometric assessment focused mainly on global publication outputs and leading contributors but lacked deeper thematic, temporal, and network analyses. Zeeshan et al. [20] provided visualizations of global research trends but emphasized outbreak‐driven surges and keyword patterns without exploring regional disparities. Furthermore, Espinoza‐Carhuancho et al. [21] limited their analysis to MPXV vaccine research, leaving out nonvaccine scientific domains and not assessing how vaccine‐related publications connect to wider MPXV research dynamics. Wang et al. [22] examined mental health publications across multiple outbreaks, including MPXV, but only from a comparative perspective, offering little insight into mpox‐specific scientific evolution. Yan and Wang [23] conducted a general bibliometric analysis using Web of Science but did not integrate genomic, spatial, or transmission‐related dimensions of research output. Shamim et al. [24] focused on pharmacological treatments and vaccines, while Mohapatra et al. [25] centered on transmission dynamics and mitigation, yet both studies provided limited evaluation of long‐term research trajectories, intercountry collaboration structures, or Africa’s contribution despite the region being historically endemic. Collective analyses of these bibliometric studies indicate that they are fragmented, topic specific, and dominated by Global North perspectives, leaving significant gaps concerning holistic research mapping, African research visibility, and a comprehensive collaboration network, gaps that the present study aims to fill.

From the foregoing, it is clear that comprehensive bibliometric investigation focused on MPXV remains limited. Our study aims to provide one of the first global bibliometric analyses of MPXV. The specific objectives of the study are (1) to provide a global trajectory of the MPXV research landscape; (2) to examine the prolific authors, institutions, and journal outlets; (3) to evaluate thematic clustering and evolution of keywords; and (4) to examine collaborative networks. Accordingly, a global bibliometric assessment of MPXV‐related publications indexed in the Scopus database was conducted. The insights derived from this study will enhance understanding of MPXV research dynamics and guide future investigations into human monkeypox infections.

## 2. Methods

### 2.1. Study Design

**FIGURE 1:**
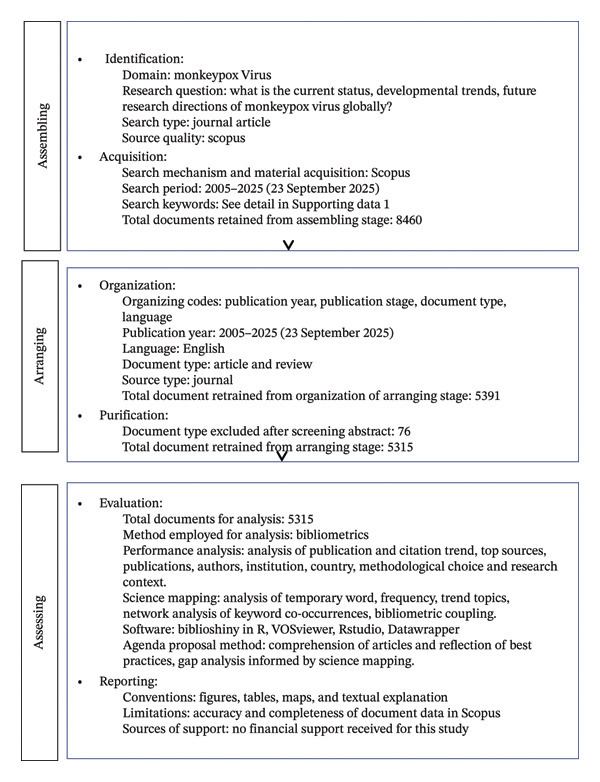
The script utilized to perform the Scopus search for the assembly and arrangement stage of the SPAR‐4‐SLR protocol.

The study design was a bibliometric study design aimed at providing a holistic global and comprehensive insight into the research landscape of MPXV. As a study design, it has been applied to diverse fields to track the evolution of studies and their progress over time [26, 27]. To ensure rigor, quality, and reproducibility, the Scientific Procedures and Rationales for Systematic Literature Review (SPAR‐4‐SLR protocol) was applied [28] (Figure 1).

### 2.2. Data Source

From the Scopus database, all MPXV studies spanning 1976–23 September 2025 were retrieved. Our choice of Scopus as a database was informed by the fact that it indexes diverse studies on multiple disciplines, including all social dimensions necessary to cover every aspect of One Health.

### 2.3. Searching Key Terms and Data Extraction

Prior to performing the search, we applied all the possible and relevant terms used to describe the virus, including “monkeypox” OR “monkey pox” OR “mpox” OR “monkeypox virus” OR “mpox virus” OR “human monkeypox” OR “orthopoxvirus monkeypox” OR “MPXV”. To obtain the final dataset, inclusion and exclusion criteria were utilized. We limited the study types to “articles” and “reviews” and language to English. Following the filtering, we downloaded the Scopus and the CSV files for further analyses. The search was conducted by two different groups of the authors, and any discrepancies were resolved via consensus as previously reported [29]. In screening the studies for inclusion, the titles, abstracts, and full‐texts as applicable were screened as previously reported, and studies not dwelling entirely on MPXV were excluded [29–31].

### 2.4. Bibliometric Analysis

To provide a comprehensive analysis, several tools were adopted, including Biblioshiny, Datawrapper, RStudio, and VOSviewer tool [32–34]. These tools were chosen because they are widely used and freely available. The trajectory of publications was plotted using a scatter plot in RStudio and fitted with a trend line along with its equation and correlation coefficient. Similarly, in RStudio, a tree map, cumulative keyword analysis, keyword evolution, a three‐field plot of countries’ research, and published journals were plotted. The utilized packages in RStudio included ggplot2, tidyverse, treemap, and wordcloud2. All network analyses were done using VOSviewer tool Version 1.6.15 for Windows (https://www.vosviewer.com). In Datawrapper, a collaboration world map was plotted to visualize the global collaborations on MPXV. All the analyses were done as previously reported [29–31, 35, 36].

## 3. Results

### 3.1. Summary of the Search

A bibliometric analysis of MPXV studies spanning 1970–2025 was conducted. As summarized in Table 1, the initial search of the Scopus database yielded 8460 MPXV publications, of which 5315 documents satisfied the inclusion criteria and were included in the final analysis. A total of 32,098 authors contributed these documents across 1547 journals. Among the final dataset, 4251 (79.98%) were original research articles, while 1064 (20.02%) were review articles. Publications on MPXV attained an average growth rate of 12.38%, with an average of 22.66 citations per document.

**TABLE 1:** Summary of the conducted search.

Description	Results
*Main information about data*	
Timespan	1976:2025
Sources (journals, books, etc.)	1547
Documents	5315
Annual growth rate (%)	12.38
Document average age	3.93
Average citations per document	22.66
References	19,704

*Document contents*	
Keywords Plus (ID)	18,494
Author’s Keywords (DE)	24,771

*Authors*	
Authors	32,098
Authors of single‐authored documents	0

*Authors Collaboration*	
Single‐authored documents	0
Coauthors per document	18.6
International coauthorships (%)	27.32

*Document types*	
Article	4251
Review	1064

### 3.2. Study Trajectory on Mpox Globally

**FIGURE 2:**
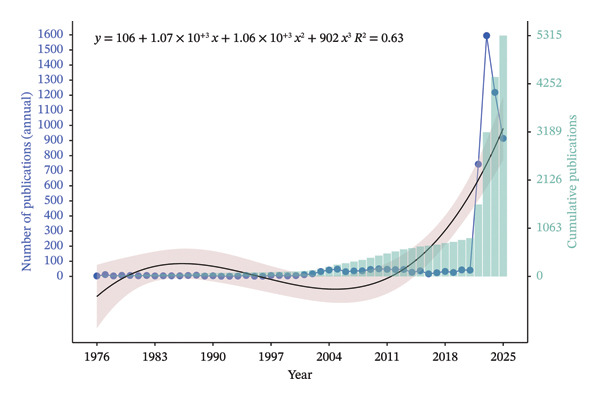
A combined bar chart fitted with scatter plot of the yearly MPXV articles published from 1976 to 2025 globally and cumulative publications. The moving light brown–colored area denotes the confidence interval. The blue dots denote the number of publications for each year. Data were extracted at 23 September 2025 from the Scopus database.

Figure 2 illustrates the publication trend on MPXV research from 1976 to 2025, highlighting the temporal progression of scientific outputs. Between 1976 and 2021, publication volumes were relatively low and steady, peaking at 50 in 2010 with a minimum of 11 publications. However, a significant spike in research output was observed in 2022 with 734, 1595, 1219, and 914 documents for 2022, 2023, 2024, and 2025, respectively. The trend line equation was *y* = −106 + 1.07 *×* 10^−3^ *x* + 1.06 *×* 10^−3^ *x* ^2^ + 902*x* ^3^, and its *R* ^2^ value was 0.63. The shaded area denotes the confidence interval, capturing the variability around the trend line.

The plot (Figure 2) indicates two distinct phases, a phase of stagnant growth and a phase of accelerated growth spanning 2022–2025. During the 40 years of stagnant growth (1976–2021), yearly publication volumes ranged from 1 to 37. A substantial surge occurred in 2022, with annual output peaking at approximately 1600 publications. Although annual publication slightly decreased to approximately 1200 in 2023 and 900 in 2024, cumulative research (teal bars) reached a total of 5315 publications by 2025.

Table 2 is a summary of the publication metrics on MPXV per year from 1976 to 2025 with their citation counts. The percentage of publication volume was generally less than 1% from 1976 to 2021, while for 2022–2025, the percentages ranged from 13.98% to 30.01%. The citation data also followed the same pattern, and the number of citations increased proportionally with publication output. The years 2022–2024 had the highest number of citations with values ranging from 7569 to 28,635.

**TABLE 2:** Number published articles and citations in MPXV per year from 1976 to 2025.

Year	Publications (%)	Total citation
1976	3 (0.06)	84
1977	12 (0.23)	244
1978	3 (0.06)	88
1979	7 (0.13)	218
1980	6 (0.11)	687
1981	4 (0.08)	64
1982	6 (0.11)	54
1983	3 (0.06)	314
1984	4 (0.08)	84
1985	4 (0.08)	422
1986	4 (0.08)	331
1987	6 (0.11)	871
1988	8 (0.15)	1237
1989	2 (0.04)	32
1990	4 (0.08)	147
1991	1 (0.02)	106
1992	1 (0.02)	27
1993	2 (0.04)	52
1994	6 (0.11)	187
1995	1 (0.02)	247
1996	1 (0.02)	18
1997	6 (0.11)	295
1998	6 (0.11)	680
1999	3 (0.06)	69
2000	5 (0.09)	133
2001	11 (0.21)	1503
2002	17 (0.32)	1388
2003	32 (0.60)	1913
2004	42 (0.79)	4145
2005	48 (0.90)	5689
2006	31 (0.58)	2291
2007	36 (0.68)	2311
2008	37 (0.70)	2850
2009	46 (0.87)	2395
2010	50 (0.94)	3558
2011	47 (0.88)	3425
2012	45 (0.85)	1757
2013	46 (0.87)	2054
2014	27 (0.51)	2121
2015	37 (0.70)	1525
2016	17 (0.32)	1186
2017	24 (0.45)	1400
2018	33 (0.62)	2489
2019	26 (0.49)	3255
2020	43 (0.81)	2810
2021	41 (0.77)	1869
2022	743 (13.98)	28,635
2023	1595 (30.01)	24,419
2024	1219 (22.94)	7594
2025	914 (17.20)	1170

Table 3 provides a summary of the top 20 most prolific authors conducting MPXV research globally and having at least of 20 publications each with their total citations. The results show a clear dominance of USA affiliated authors with 14 out of the 20 most prolific authors. Other countries of the top 20 authors were Colombia, Germany, India, Nepal, and Russia with one prolific author each. The dominance of the USA indicates that it plays a prominent role in advancing MPXV research globally. The top seven authors were from the USA, and these were affiliated to either Emory University or National Center for Emerging and Zoonotic Infectious Diseases or Centers for Disease Control and Prevention (NCEZID). The top three authors were Inger K. Damon (47 publications, 5890 citations), Mary G. Reynolds (39 papers, 4721 citations), and Victoria A. Olson (35 papers, 3431 citations).

**TABLE 3:** Details of authors who published at least 20 MPXV‐related articles globally.

Rank	Authors	Articles (%)	Fractionalized frequency	Citations	Institution	Country
1	Damon, Inger K.	47 (0.88)	10.34	5890	Emory University	USA
2	Reynolds, Mary G.	39 (0.73)	9.00	4721	National Center for Emerging and Zoonotic Infectious Diseases	USA
3	Olson, Victoria A.	35 (0.66)	8.05	3431	Centers for Disease Control and Prevention	USA
4	Hughes, Christine Marie	33 (0.62)	7.90	3055	National Center for Emerging and Zoonotic Infectious Diseases	USA
5	Mccollum, Andrea M.	30 (0.56)	7.77	3396	Centers for Disease Control and Prevention	USA
6	Karem, Kevin L.	29 (0.55)	6.28	2143	Centers for Disease Control and Prevention	USA
7	Carroll, Darin S.	28 (0.53)	5.09	1734	Centers for Disease Control and Prevention	USA
8	Shchelkunov, Sergey N.	28 (0.53)	5.00	1592	SRCVB Vector	Russia
9	Dhama, Kuldeep	25 (0.47)	5.00	378	Indian Veterinary Research Institute	India
10	Doty, Jeffrey B.	24 (0.45)	4.70	1973	Centers for Disease Control and Prevention	USA
11	Mazzotta, Valentina	24 (0.45)	4.64	720	IRCCS Istituto Nazionale Malattie Infettive Lazzaro Spallanzani	Italy
12	Moss, Bernard A.	23 (0.43)	4.48	1133	National Institute of Allergy and Infectious Diseases	USA
13	Rodriguez‐Morales, Alfonso J.	23 (0.43)	4.45	548	Fundación Universitaria Autónomade las Américas	Colombia
14	Hutson, Christina L.	22 (0.41)	4.38	1316	Centers for Disease Control and Prevention	USA
15	Nakazawa, Yoshinori J.	22 (0.41)	4.35	2014	National Center for Emerging and Zoonotic Infectious Diseases	USA
16	Nitsche, Andreas	22 (0.41)	4.32	641	Leibniz Research Centre for Working Environment and Human Factors	Germany
17	Sah, Ranjit Kumar	22 (0.41)	4.18	576	Tribhuvan University	Nepal
18	Hruby, Dennis E.	21 (0.40)	4.11	1313	SIGA Technologies Incorporated	USA
19	Jährling, Peter B.	20 (0.38)	4.01	1639	National Institutes of Health	USA
20	Satheshkumar, Panayampalli Subbian	20 (0.38)	3.98	634	Centers for Disease Control and Prevention	USA

### 3.3. Most Prolific Authors and Affiliations on MPOX

### 3.4. Top Funders of MPXV Studies

In addition to the prolific authors, we also evaluated the top ten funders of MPXV research globally, as summarized in Table 4. Among the funders, the National Natural Science Foundation of China (NSFC) ranked first, appearing in 297 publications (5.59%). This was followed funders in the USA, including the National Institute of Health (5.31%), the National Institute of Allergy and Infectious Diseases (4.89%), the Centers for Disease Control and Prevention (2.14%), and the National Science Foundation (0.79%). European funding organizations were also influential, led by the European Commission (1.51%) and the Horizon 2020 Framework Programme (0.85%), while the United Kingdom made notable contributions through UK Research and Innovation (1.05%) and the Wellcome Trust (0.92%).

**TABLE 4:** Top 10 funders or sponsors of MPXV research between 1976 and 2025 globally.

Rank	Funding sponsor	Country	Documents (%)
1	National Natural Science Foundation of China	China	297 (5.59)
2	National Institutes of Health	USA	282 (5.31)
3	National Institute of Allergy and Infectious Diseases	USA	260 (4.89)
4	National Key Research and Development Program of China	China	203 (3.82)
5	Centers for Disease Control and Prevention	USA	114 (2.14)
6	European Commission	Europe	80 (1.51)
7	UK Research and Innovation	UK	56 (1.05)
8	Wellcome Trust	UK	49 (0.92)
9	Horizon 2020 Framework Programme	Europe	45 (0.85)
10	National Science Foundation	USA	42 (0.79)

### 3.5. Most Cited Papers on MPXV Globally

A summary of the top 20 most cited publications is presented in Table 5, providing valuable insights into the intellectual structure and research influence within the field. The top twenty most cited publications span from 1987 to 2022, highlighting both the lasting impact of early seminal studies and the recent surge in research activity. The highest‐cited paper, authored by Thornhill et al. [37], was published in *The New England Journal of Medicine* and it accumulated 1550 citations, averaging 387.5 citations per year. Likewise, the works of Bunge et al. [38] in *PLOS Neglected Tropical Diseases* and Adler et al. [39] in *The Lancet Infectious Diseases* achieved exceptionally high citation rates, averaging over 230 citations annually, underscoring the intensified global focus on the topic in recent years. Earlier studies, such as those by Jezek et al. [40] and Fine et al. [41], continue to attract citations, though at a much lower rate (< 15 citations per year). Meanwhile, publications from the 2000s, including Reed et al. [4], Likos et al. [42], and McFadden [43], have sustained moderate citations. Prominent journals specializing in infectious disease research, such as *The New England Journal of Medicine*, *The Lancet Infectious Diseases*, *Clinical Infectious Diseases*, and *Nature Medicine*, were prominently represented among the top‐cited publications.

**TABLE 5:** Most cited papers on MPXV globally with their citation metrics.

Articles	DOI	Total citations	Total citations per year
Thornhill et al. [37], New England Journal of Medicine	10.1056/NEJMoa2207323	1550	387.5
Bunge et al. [38], PLoS Neglected Tropical Diseases	10.1371/journal.pntd.0010141	1273	318.2
Adler et al. [39], The Lancet Infectious Diseases	10.1016/S1473‐3099 (22)00228–6	945	236.2
Galdiero et al. [44], Molecules	10.3390/molecules16108894	846	56.4
Mccollum and Damon [45], Clinical Infectious Diseases	10.1093/cid/cit703	802	66.8
Reed et al. [4], New England Journal of Medicine	10.1056/NEJMoa032299	690	31.4
Rimoin et al. [46], Proceedings of the National Academy of Sciences of the United States of America	10.1073/pnas.1005769107	661	41.3
Yinka‐Ogunleye et al. [47], The Lancet Infectious Diseases	10.1016/S1473‐3099 (19)30294–4	643	91.9
Likos et al. [42], Journal of General Virology	10.1099/vir.0.81215‐0	631	30.0
Di Giulio and Eckburg [48], The Lancet Infectious Diseases	10.1016/S1473‐3099 (03)00856–9	601	27.3
Alakunle et al. [49], Viruses	10.3390/v12111257	589	98.2
Isidro et al. [50], Nature Medicine	10.1038/s41591‐022–01907‐y	581	145.2
Petersen et al. [51], Infectious Disease Clinics of North America	10.1016/j.idc.2019.03.001	544	77.7
Tarín‐Vicente et al. [52], Lancet	10.1016/S0140‐6736 (22)01436–2	530	132.5
Jezek et al. [40], Journal of Infectious Diseases	10.1093/infdis/156.2.293	511	13.1
Beer and Rao [53], PLoS Neglected Tropical Diseases	10.1371/journal.pntd.0007791	492	70.3
Huhn et al. [54], Clinical Infectious Diseases	10.1086/498,115	480	22.9
Fine et al. [41], International Journal of Epidemiology	10.1093/ije/17.3.643	471	12.4
Mcfadden [43], Nature Reviews Microbiology	10.1038/nrmicro1099	465	22.1
Sklenovská and Van Ranst [55], Frontiers in Public Health	10.3389/fpubh.2018.00241	463	57.875

**FIGURE 3:**
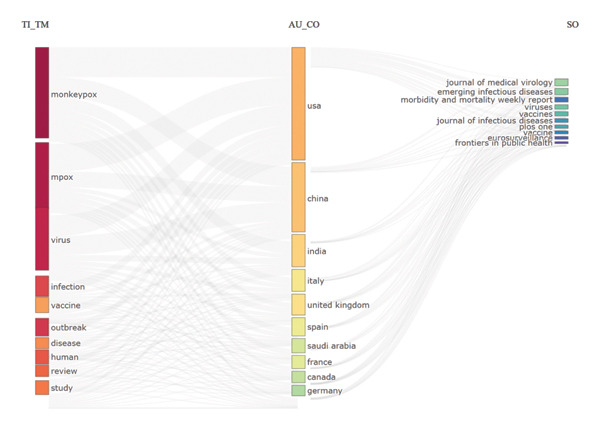
Three‐field plot of countries’ research and published journals. Left field = title keywords/themes (TI_TM), central field = country (AU_CO), and right field = source titles (SO).

Figure 3 depicts the interrelationships among research themes, contributing countries, and publishing journals in Mpox‐related studies. On the left, the most frequently occurring keywords: “monkeypox,” “mpox,” “virus,” “infection,” “vaccine,” “outbreak,” “disease,” “human,” “review,” and “study” capture the primary thematic areas of focus. These keywords indicate that MPXV research has largely concentrated on viral biology, transmission dynamics, vaccine development, and outbreak management. The central section highlights the major contributing countries, with the USA leading, followed by China, India, Italy, and the United Kingdom. Other active contributors include Spain, Saudi Arabia, France, Canada, and Germany, reflecting the global and collaborative nature of MPXV research. The prominence of the USA and China underscores their key roles in driving scientific output and outbreak response. On the right, the principal publishing platforms include the *Journal of Medical Virology*, *Emerging Infectious Diseases*, *Morbidity and Mortality Weekly Report*, *Viruses*, *Vaccines*, *Journal of Infectious Diseases*, *PLOS One*, *Eurosurveillance*, and *Frontiers in Public Health*. These journals primarily emphasize infectious disease epidemiology, virology, and public health research.

**FIGURE 4:**
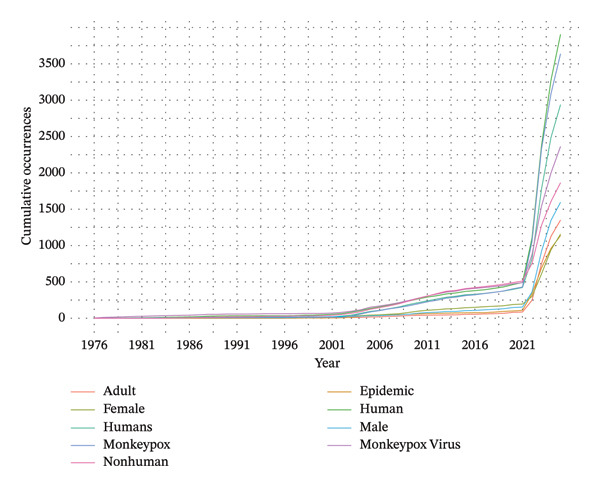
A trend line plot of the cumulative top nine keywords used in MPXV research globally from 1976 to 2025. Each color represents a keyword.

Figure 4 shows the cumulative frequency of the top nine keywords associated with MPXV research from 1976 to 2025. It summarizes the cumulative frequencies of these keywords over the years. The top nine keywords were “monkeypox,” “monkeypox virus,” “humans,” “nonhuman,” “human,” “epidemic,” “adult,” “male,” and “female.” The cumulative usage of these keywords remained low until 2003. Similarly, the cumulative usage also surged after 2021.

**FIGURE 5:**
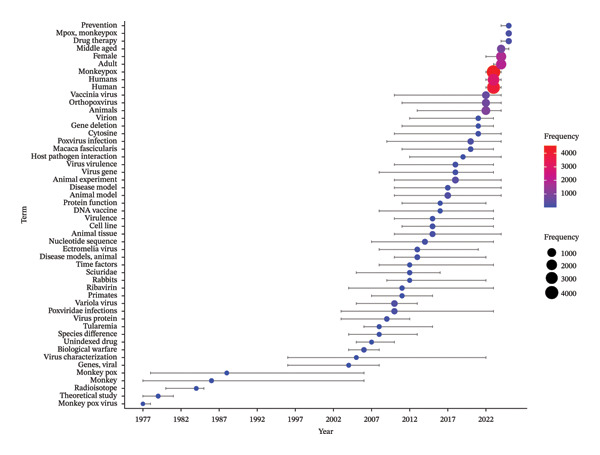
A temporal evolution of the various keywords used in MPXV research between 1976 and 2025. The color legends depict the frequencies of usage. The blue dot and the straight line across its diameter depict a particular keyword and its evolution or usage over time.

Figure 5 depicts the temporal evolution and frequency of key research terms associated with MPXV studies from 1976 2025. The most used keywords were “human,” “humans,” and “monkeypox” as indicated by their higher frequencies compared to other keywords. The earliest recorded keyword was “monkeypox virus” appearing in 1977. Other early keywords include “theoretical study,” “radioisotope,” “monkey,” and “monkeypox.” In terms of spread, the top three most used keywords were “monkey,” “monkeypox” and “virus characterization” with usage span that were 1977–2006, 1978–2006, and 1996–2022, respectively. From 2022 onward, newer keywords have evolved, including “prevention,” “mpox,” “monkeypox,” “drug therapy,” “female,” “adult,” and “humans.”

As further illustrated using the tree map (Supporting data 2), various keywords have been used differently in MPXV research globally. The term “monkeypox” appears most frequently, with 5913 mentions (13.46%), followed by “human” (8.95%) and “humans” (6.69%). Other keywords were “male” (5.78%), “adult” (4.39%), “female” (4.11%), “nonhuman” (4.25%), “vaccination” (3.24%), “controlled study” (2.38%), “epidemic” (2.67%), “review” (2.05%), and “public health” (1.94%), “smallpox” (2.2%), and “smallpox vaccine” (2.86%).

**FIGURE 6:**
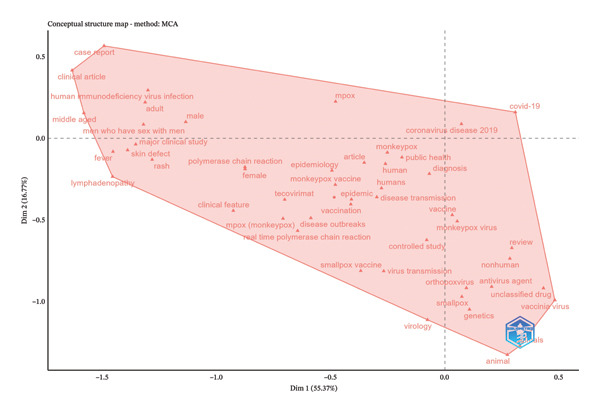
A factorial analysis of the different keywords used in MPXV research showing two dimensions. The six‐sided figure encompasses the majority of the keywords utilized in mpox research and span over both dimensions.

The thematic cluster of the keywords is captured in the multiple correspondence analysis (MCA), also called factorial analysis (Figure 6). The upper‐left cluster includes terms such as “case report,” “clinical article,” “human immunodeficiency virus infection,” “fever,” “rash,” “lymphadenopathy,” and “men who have sex with men.” The left and bottom part of Dimension 1 comprises keywords, including “monkeypox,” “mpox,” “epidemic,” “vaccination,” “epidemiology,” “polymerase chain reaction,” “tecovirimat,” and “disease transmission.” The right/lower cluster includes keywords such as “smallpox,” “vaccinia virus,” “orthopoxvirus,” “genetics,” “antiviral agent,” “animal,” and “COVID‐19.” Figure 7(a) indicates the clustering of the various keywords used in MPXV research globally. The clustering revealed three thematic areas: vaccination, human subjects, and the virus.

FIGURE 7(a) Keyword co‐occurrence network analysis of all keywords used in MPXV research globally. The circular bubbles indicate the size of keywords usage, while the lines and their thickness indicate strength of their usage. The different colored keywords indicate different clusters, totaling four in number. (b) Bibliographic coupling sources/network visualization mapping based on the number of citations. (c) Mapping of the top 20 leading collaborating institutions in MPXV research between 1976 and 2025 based on bibliometric coupling.

**Figure:**
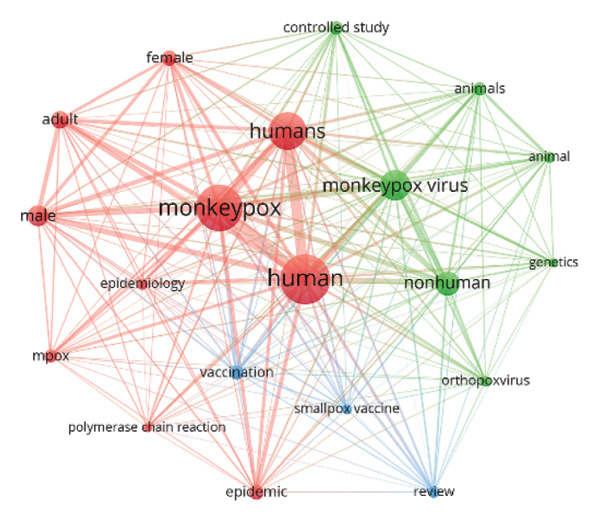
(a)

**Figure:**
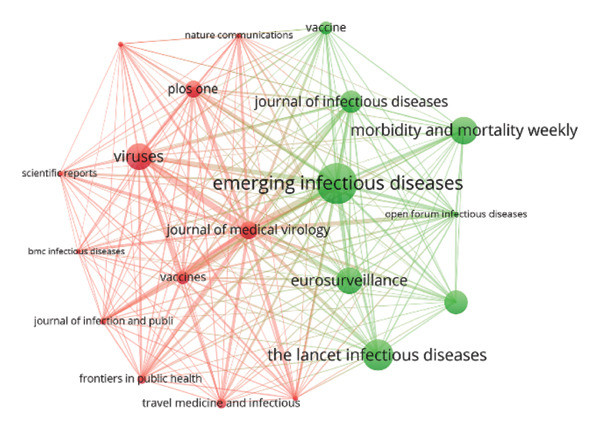
(b)

**Figure:**
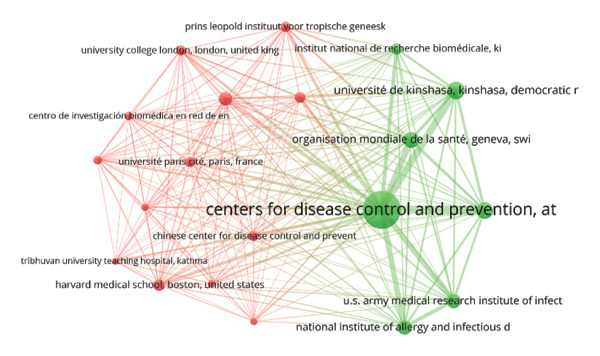
(c)

### 3.6. Journal Outlets and the Co‐occurrence Network

In addition to the prolific authors, their affiliations, and keywords, top journal destinations and their collaborations were also analyzed. Table 6 is a summary of the top 20 journals publishing MPXV‐related research, highlighting their publication output, citation impact, and quality metrics (impact factor and quartile ranking). The *Journal of Medical Virology* published the highest number of MPXV‐related papers (132; 2.48%), followed closely by *Emerging Infectious Diseases* (123; 2.31%) and *Viruses* (115; 2.16%). The most cited journals include *Emerging Infectious Diseases* (5163 citations), *Morbidity and Mortality Weekly Report* (3429), and *Viruses* (3321). Furthermore, a significant proportion of the leading journals fall within the Q1 and Q2 quartiles. High‐impact journals such as *Lancet Infectious Diseases* (IF = 31, Q1) and *Nature Communications* (IF = 15.7, Q1) were also prominent. Figure 7(b) is a journal cocitation network map showing how often journals are cited together in monkeypox research literature. “*Emerging Infectious Diseases*” is the most central and frequently cocited journal (largest green node), indicating it plays a pivotal role in the dissemination of MPXV research.

**TABLE 6:** Details of journals that published top 20 MPXV‐related articles.

Journals	Articles (%)	Citations	IF (2024)^∗^	Q ranking^∗^
Journal of Medical Virology	132 (2.48)	2220	4.6	Q1
Emerging Infectious Diseases	123 (2.31)	5163	6.6	Q1
Viruses	115 (2.16)	3321	3.5	Q2
Vaccines	97 (1.83)	1557	3.4	Q2
PLOS One	70 (1.32)	2103	2.6	Q2
Journal of Infectious Diseases	66 (1.24)	2782	4.5	Q2
Vaccine	66 (1.24)	1566	3.5	Q2
Morbidity and Mortality Weekly Report	62 (1.17)	3429	17.3	Q1
Eurosurveillance	59 (1.11)	3315	7.8	Q1
Frontiers in Public Health	52 (0.98)	1076	3.4	Q1
Journal of Infection and Public Health	52 (0.98)	787	4	Q1
Scientific Reports	48 (0.90)	547	3.9	Q1
Travel Medicine and Infectious Disease	44 (0.83)	1198	4.7	Q1
Open Forum Infectious Diseases	43 (0.81)	597	3.8	Q2
Virology	41 (0.77)	2512	2.3	Q3
Pathogens	40 (0.75)	694	3.3	Q2
Journal of Virology	39 (0.73)	2788	3.8	Q2
Antiviral Research	38 (0.71)	1449	4	Q1
Nature Communications	38 (0.71)	573	15.7	Q1
Lancet Infectious Diseases	36 (0.68)	3911	31	Q1

^∗^Impact factor (IF) and Q ranking based on the Journal Citation Reports (JCR) 2024—released in June 2025.

### 3.7. Institutions and Global Collaboration

We analyzed various aspects of top institutions, countries, and collaborative networks. Figure 7(c) shows institutional collaborations in MPXV research. The Centers for Disease Control (CDC), USA, is the largest and most central node, indicating it is the top collaborative institution in monkeypox research, often partnering with many others globally. Two main collaborative clusters are visible: Green Cluster (USA‐led collaborations), which includes the CDC, WHO, Université de Kinshasa (DRC), & USA Army Medical Research Institute, and the NIH/NIAID. This reflects a strong partnership between USA, African institutions, and global health organizations. Red Cluster (European and Asian collaborators) includes Institut Pasteur (France), Chinese CDC, Harvard Medical School, University College London, and focuses on academic and international research collaborations, particularly from Europe, Asia, and Latin America. The dense lines (edges) in the clusters reflect frequent coauthorship, indicating robust global collaboration.

Table 7 summarizes the top institutions contributing to MPXV research (with ≥ 40 publications). The USA leads MPXV research output and influence. The CDC ranks first with 192 publications (3.61%) and the highest citation count (10,329). Other USA institutions such as the National Institute of Allergy and Infectious Diseases (NIAIDs), the USA Army Medical Research Institute of Infectious Diseases (USAMRIID), and NCEZID also appear prominently. The WHO/OMS, Switzerland, ranks third with 86 publications (1.62%). The Université de Kinshasa (Congo) with 49 papers and 1836 citations, is the only African institution in the top 10.

**TABLE 7:** The leading institutions in MPXV research with at least 40 published articles.

Rank	Institutions	Documents (%)	Total global citations
1	CDC, USA	192 (3.61)	10,329
2	NIAID, USA	86 (1.62)	5370
3	OMS, Switzerland	86 (1.62)	9053
4	USAMRIID, USA	85 (1.60)	6779
5	NCEZID, USA	67 (1.26)	5453
6	Harvard Medical School, USA	62 (1.17)	1891
7	UKHSA, UK	56 (1.05)	2297
8	LSHTM, UK	50 (0.94)	2872
9	Université de kinshasa, Congo	49 (0.92)	1836
10	China CDC, China	48 (0.90)	584
11	University College London, UK	46 (0.87)	2954
12	UCAS, China	45 (0.85)	463
13	SRCVB VECTOR, Russia	44 (0.83)	1924
14	University of Oxford, UK	41 (0.77)	602

Between 1976 and 2025, MPXV research was dominated by contributions from a few key countries (Table 8). The USA led significantly, producing 1879 publications (35.35%) and achieving the highest citation count (62,272). China ranked second with 687 documents (12.93%), followed by India (518; 9.75%). Both countries show strong emerging research engagement though with comparatively lower citation counts (7985 and 5,747, respectively). The United Kingdom was fourth, contributing 463 publications (8.71%) but standing out with a relatively high citation impact (16,618), indicating the quality and visibility of its research.

**TABLE 8:** A summary of the top 10 countries involved in MPXV research between 1976 and 2025.

Rank	Country	Documents (%)	Total global citations
1	United States	1879 (35.35)	62,272
2	China	687 (12.93)	7985
3	India	518 (9.75)	5747
4	United Kingdom	463 (8.71)	16,618
5	Saudi Arabia	277 (5.21)	4798
6	Italy	259 (4.87)	7506
7	Germany	221 (4.16)	9923
8	Spain	214 (4.03)	6778
9	Canada	212 (3.99)	7493
10	France	195 (3.67)	7051

Figure 8(a) shows a global map of research publications on the monkeypox virus from 1976 to 2025. The USA was the top contributor (deep blue, largest bubble), indicating it has published the most on monkeypox within this period. The United Kingdom, Germany, India, China, and Russia also had high publication counts (large blue circles), suggesting strong research output. Countries across Europe, South America, and parts of Asia and Africa (e.g., Brazil, Nigeria, South Africa, and Egypt) have moderate publication activity (orange/red circles). Several countries in central Africa, Central Asia, and parts of South America have small or no circles, indicating minimal or no published research.

FIGURE 8(a) Total publications per country worldwide on MPXV. The circular legend indicates the various publications from each of the countries. (b) Intercountry collaborations on MPXV research globally. Size of the circular bubbles indicates the size of the collaborations. The links (lines) indicate the various collaborations between the various countries.

**Figure:**
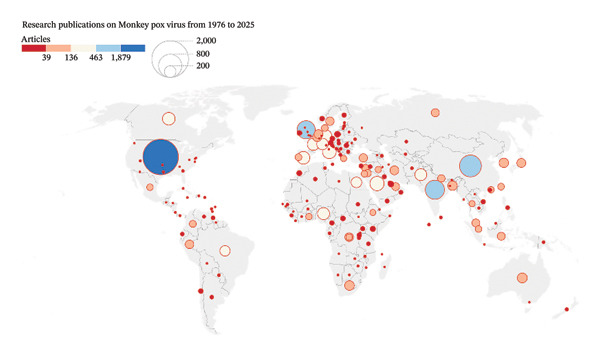
(a)

**Figure:**
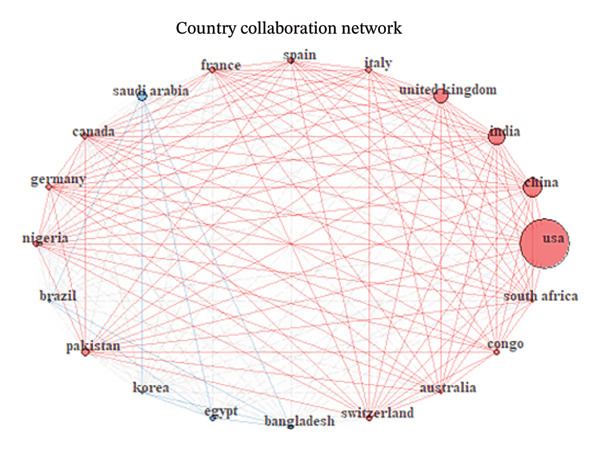
(b)

Figure 8(b) shows country collaboration networks on MPXV research, visualizing how countries coauthor or collaborate on research publications. The USA has the largest node, indicating that it is the most active country in international collaborations. Other significant collaborators, including China, India, and the United Kingdom, are represented by relatively larger red nodes. Countries such as Bangladesh, Egypt, and Korea have smaller nodes and fewer connections, indicating limited but present involvement. The USA sits at the core, driving much of the research and forming collaborative bridges between regions.

## 4. Discussion

This bibliometric study aimed to provide a global perspective of the MPXV trends, productivity, and collaboration patterns from 1976 to 2025. We observed two phases of scientific growth on MPXV, a period of stagnant and accelerated growths, pre and post the global outbreak, respectively. Overall, the volume of studies revealed that scientific interest in MXPV has evolved slowly over 4 decades but surged exponentially following the 2022 global outbreak [56, 57]. Compared with an earlier study that evaluated the volume of MPXV studies globally (2001–2021), which reported 507 studies on MPXV [20], which is three times less than our findings. Similarly, the volume of studies in previous bibliometric reports on MPXV were lower than the reported number of publications in our study [19, 21, 23–25, 58]. The difference in the volume of studies could be attributed to the different search terms used in their study and the fact that previous studies considered aspects of the MPXV.

However, the observed volume in our study is comparable to the volume of studies in other fields, including vaccine uptake, human immunodeficiency virus and acquired immunodeficiency syndrome (HIV/AIDS) in Nigeria, and avian influenza virus (AIV) globally. The sharp rise in the volume of studies observed between 2022 and 2023 coincides with the declaration of a global health emergency by the WHO [59]. The temporal trend demonstrates that MPXV‐related research remained minimal for more than 4 decades, with less than 50 publications per year until 2021. This pattern aligns with previous reports that indicate changes in major events following major epidemics and pandemics such as observed during AIV during various global waves [29]. The period of low research output, from 1976 to 2021, was longer than the early days observed in previous bibliometric studies on vaccine uptake in Africa [60], AIV [29], and HIV/AIDS [61]. Put together, the analysis of the study trajectory indicates an increasing trend in the volume of studies, driven with major events such as reported outbreaks from 2001 and the global outbreak of 2022. There is a need to conduct and sustain studies on MPXV so that all aspects of the biology of the virus can be fully understood.

Our analysis of the study types indicates a dominance of research articles over reviews in a ratio of 4 (primary research):1 (review articles). This aligns with a previous bibliometric study on MPXV that reported 399 (79.64%) primary studies and 108 (20.36%) review papers [20]. The constant ratio indicates that the rate of synthesis of original papers to reviews has remained the same pre and post the global outbreak. It also indicates that there is a strong empirical orientation within the field. Reviews, especially systematic reviews, when well written, serves as evidence that can inform policies and also reveal gaps for future studies [62, 63].

Authorship analysis revealed a total of 32,098 authors involved in the various studies. Our analysis of the top twenty authors revealed that they published at least 537 articles, representing approximately 10% of the total studies. This aligns with previous bibliometric studies that also indicated the top 10–20 authors being responsible for at least 10%–20% of the total studies in a field [29, 60]. These findings indicate that their contributions are very influential in the field. In our study, authors from the USA dominated, particularly through the CDC, the NIAID, and the USAMRIID. Of the 20 most prolific authors, 70% came from the USA, while the rest of the authors were from Colombia, Germany, India, Nepal, and Russia. The dominance of the USA, a high income country, is in line with previous reports on other pathogens, including HIV/AIDS, Lassa fever virus, and AIV [29, 61, 64].

The dominance of the USA indicates its prominence in conducting MPXV studies. Furthermore, the mixture of high‐income and resource‐poor countries indicates interest among diverse nations probably driven by exported cases and the global outbreak of 2022 [65]. This is in contrast to a previous bibliometric study that examined vaccine uptake in Africa, where it was noted that African countries dominated the top nations conducting studies on vaccine uptake in the region [60]. The dominance of USA and the clear absence of Africa countries, where the virus is considered endemic, is call for concern. These disparities could be explained by the presence of inadequate research infrastructure [66]. Despite these disparities, the top institutes/universities contributed a substantial portion of the total research output and citations. Their leadership highlights the strategic role of USA federal agencies in the investigations of diseases and in global health coordination. The top five authors have dominated the field pre and post the global pandemic [20].

To further explain the disparity, we examined the top funders of MPXV research globally. The spread of the funders also showed geographical disparities. The NSFC and USA funding agencies, such as the NIH, NIAID, and CDC, were the most prominent sponsors. European bodies, including the European Commission, Horizon 2020, and the Wellcome Trust, also featured prominently. The dominance of funders from high‐income countries reflects both resource availability and geopolitical priorities in global health research investment. This aligns with previous reports that also highlighted the same disparity [67]. However, it also exposes disparities in research infrastructure between endemic regions and the global North [68]. Furthermore, our analysis of the funders further shows the absence of prolific funders from endemic regions, including West and Central Africa, implying that authors from these regions also have to compete with foreign authors for international funding calls. There is a need for localized funding in endemic countries to support MPXV studies.

The most cited studies show robust evidence on the evolving epidemiology, clinical features, virology, and public health significance of human monkeypox. Large multicountry outbreak investigations demonstrate a clear shift from a historically rare zoonotic disease confined to Central and West Africa to sustained human‐to‐human transmission in nonendemic regions, particularly during the 2022 global outbreak [37, 39, 52]. Clinical studies consistently describe fever, lymphadenopathy, and vesiculopustular rash, while more recent outbreaks report atypical presentations, including localized genital and perianal lesions, suggesting changes in transmission patterns and exposure routes [37, 39]. Epidemiological analyses highlight the long‐term consequences of discontinued smallpox vaccination, linking declining population immunity to the resurgence and expansion of MPXV across multiple regions [40, 46, 53]. Genomic and phylogenetic investigations reveal evidence of viral microevolution, with distinct clades circulating globally and mutations that potentially enhance adaptation to human hosts, although increased virulence has not been conclusively demonstrated [50, 69]. Reviews further emphasize the roles of close physical contact, healthcare exposure, and social networks, spillover from reservoir rodents in sustaining transmission chains [38, 70]. Importantly, public‐health‐focused studies underscore persistent gaps in surveillance, diagnostics, and equitable access to vaccines and therapeutics, particularly in endemic African countries where MPXV has circulated for decades [53]. Overall, the evidence from the most cited studies positions MPXV as a re‐emerging global health threat that necessitates integrated surveillance, genomic monitoring, targeted vaccination strategies, and strengthened international collaboration.

Keyword analysis is an integral aspect of bibliometric studies as it can reveal the evolution of the keywords as well as their thematic clustering [71]. The number of keywords in our study stood at 24,771, indicating the field has evolved strongly with studies covering diverse areas. Cumulative analysis of top keywords revealed three distinct phases of MPXV research. First, a period of slow growth starting from 1976 to 2000, 2001 to 2021, and 2022 to 2025. The 1976–2001 phase of keyword growth coincides with the period the virus was tagged endemic to West and Central Africa [72], while the 2001–2021 phases coincide with the period exported cases of the virus began to emerge globally, and the third phase of growth coincides with the global outbreak of the virus [65]. During these phases, keywords related to humans and the virus dominated the research landscape, indicating a dominant focus on the virus and its effects on human populations, with increasing attention to host dynamics and gender‐based differences. From 2022, newer keywords such as “prevention,” “mpox,” “monkeypox,” “drug therapy,” “female,” “adult,” and “humans,” indicating an emphasis on prevention of virus and therapy following the 2022 global outbreak [73]. Conceptual cluster analysis highlighted three main thematic areas: (1) clinical and demographic studies emphasizing HIV coinfection and human case profiles; (2) epidemiological and diagnostic research focusing on outbreak dynamics and vaccine evaluation; and (3) molecular and virological studies linking MPXV with other orthopoxviruses. The research landscape of the virus indicates a clear evolution of keywords over time. The absence of endemic countries among the keywords indicates low productivity of research among the endemic countries [74]. Also, keywords centered on phylodynamics and phylogeography, which when applied in these studies, sought to understand the spread of the virus across settings are prominently missing even though a few of such studies already exist; their coverage is limited. Setting up such studies would enhance our understanding of the transmission dynamics of the virus.

Preferred journal destinations indicate preference of authors for these journals. This preference aligns with previous bibliometric studies showing that authors often select certain journals as their preferred outlets [29, 60, 61]. Top journal destinations showed the presence of core area, specialty, and multidisciplinary journal outlets, showing diverse studies, as these journals have focal areas that define their scope. Several factors are usually taken into consideration by authors when deciding journal destination for their studies, including ranking, impact factor, and visibility of their studies, peer‐review quality, and rigor [75]. Visualization network analysis of the journal destinations highlighted the dominance of *Emerging Infectious Diseases* as the most influential and cocited journal outlet within the journal destination network.

Our analysis of the top institutions and collaboration showed some interesting findings. First, it showed a dominance of institutions based in resource‐rich countries over resource‐poor settings, further aligning with the disparity observed earlier among the prolific authors. The income‐rich nations were dominated by countries such as China and USA, and this aligns with previous reports [20, 29]. Their dominance indicates leadership in the fight against infectious disease agents, including MPXV virus. The involvement of the WHO/OMS, Switzerland, highlights its pivotal role in coordinating international research and public health response. The ranking of the Université de Kinshasa (Congo) among the top 10 reflects their central role in the fight against MPXV in Central Africa and the growing research capacity in the region, indicating that it has assumed a leadership role in the fight against the virus. Our global collaboration networks showed that the USA serves as the central node in international partnerships, maintaining strong research ties with China, the United Kingdom, India, and Australia. These collaborations have intensified post‐2022, as reflected by dense coauthorship networks and multinational studies. However, regions such as Central and West Africa, where MPXV remains endemic [3], show relatively low research participation, indicating a persistent geographic gap. This imbalance emphasizes the need for capacity building, data sharing, and sustained investment in local research systems. Effective management of MPXV outbreaks demands robust international collaboration [76]. Global health agencies, national governments, and nongovernmental organizations (NGOs) must work together to provide financial, technical, and research assistance to countries most affected by the disease. Through coordinated global partnerships, the development of new vaccines and therapeutics can be expedited, while public health strategies can be refined to more effectively address MPXV and other emerging infectious threats [3].

### 4.1. Limitations of the Study

Our study has several limitations. First, the search was limited to a single database, Scopus. Second, only document that were either articles or reviews were included. Third, only studies written in English were included in the final dataset. As a result, we may have excluded studies not indexed in Scopus or not written in English.

## 5. Conclusion

This bibliometric analysis reveals that MPXV research has transitioned from a niche tropical disease focus to a global public health priority, receiving attention from global and regional health institutions. The field is characterized by strong international collaboration but with an uneven geographic representation, with dominance by institutions in the USA, Europe, and China. Research output and citations have grown exponentially since the 2022 outbreak, reflecting heightened scientific and policy interest. However, endemic regions in Africa remain underrepresented in authorship, funding, and institutional leadership. High‐impact publications in recent years have redefined the scientific landscape, driving innovation in diagnostics, therapeutics, and epidemiological modeling. Nevertheless, the thematic structure suggests that, while foundational knowledge is well established, there is a need for more balanced, regionally inclusive, and mechanistically focused research. Strengthening collaborative networks, enhancing funding equity, and integrating genomic, clinical, and ecological perspectives will be critical to advancing MPXV research and preparedness for future orthopoxvirus outbreaks.

## Author Contributions

Conceptulization: Uwem Okon Edet and Md Zulfekar Ali. Methods, software, and validation: Md Zulfekar Ali, Uwem Okon Edet, and Clement Meseko. Screening of studies: Md Zulfekar Ali, Uwem Okon Edet, Bassey Ini Ubi, Edema Enogiomwan Imalele, Bassey Edet, Francisca Nwaokorie, and Clement Meseko. Supervision: Md Zulfekar Ali and Clement Meseko. Original drat: Uwem Okon Edet and Md Zulfekar Ali. Review and editing: Uwem Okon Edet, Md Zulfekar Ali, Bassey Ini Ubi, Edema Enogiomwan Imalele, Bassey Edet, Francisca Nwaokorie, and Clement Meseko.

## Funding

No funding was received for this study.

## Disclosure

All authors have approved the study.

## Ethics Statement

Ethical approval is not necessary for this study as it is a bibliometric study based on published literature.

## Conflicts of Interest

The authors declare no conflicts of interest.

## Supporting Information

Additional supporting information can be found online in the Supporting Information section.

## Supporting information


**Supporting Information 1** Supporting data 1: Scopus Search advance keywords.

**Supporting Information 2** Supporting data 2: A Tree map representation of keywords plus (top 30) based on frequency of usage. Each box represents a keyword, the number of usages of the keywords, and the frequency of usage of these keywords.

## Data Availability

The data that support the findings of this study are available from the corresponding authors upon reasonable request.
