# Supplementary material for: First Comprehensive Global Bibliometric Analysis of Monkeypox Virus Research Landscape From 1976 to 2025
Source: Can J Infect Dis Med Microbiol. 2026 May 24;2026:2362823. doi: 10.1155/cjid/2362823 (PMC13199685; doi:10.1155/cjid/2362823)
Supplement: Supplementary file 1 — Supporting Information 1 Supporting data 1: Scopus Search advance keywords. [file CJID-2026-2362823-s001.docx]

**Supplementary data 1: Scopus Search advance keywords**

TITLE-ABS-KEY ( "monkeypox" OR "monkey pox" OR "mpox" OR "monkeypox virus" OR "mpox virus" OR "human monkeypox" OR "orthopoxvirus monkeypox" OR "hMPXV" OR "MPXV" ) AND PUBYEAR > 1974 AND PUBYEAR < 2026 AND ( LIMIT-TO ( DOCTYPE , "ar" ) OR LIMIT-TO ( DOCTYPE , "re" ) ) AND ( LIMIT-TO ( LANGUAGE , "English" ) ) AND ( LIMIT-TO ( PUBSTAGE , "final" ) )
