# Supplementary material for: First Comprehensive Global Bibliometric Analysis of Monkeypox Virus Research Landscape From 1976 to 2025
Source: Can J Infect Dis Med Microbiol. 2026 May 24;2026:2362823. doi: 10.1155/cjid/2362823 (PMC13199685; doi:10.1155/cjid/2362823)

**Supplementary data 2:** A Tree map representation of keywords plus (top 30) based on frequency of usage. Each box represents a keyword, the number of usages of the keywords and the frequency of usage of these keywords.


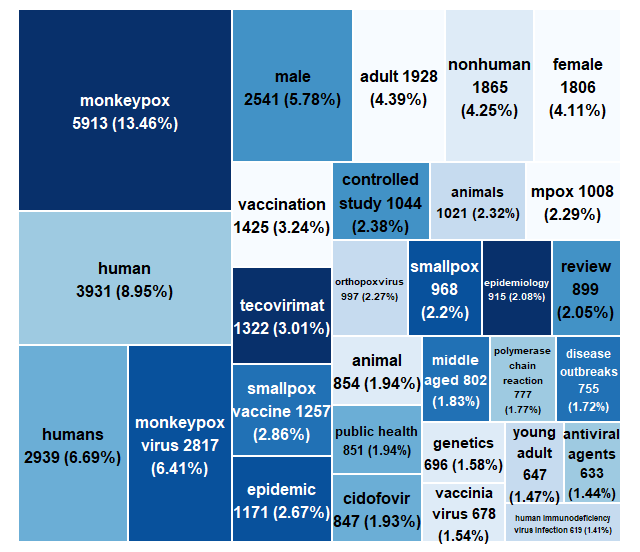

Supplement: Supplementary file 2 — Supporting Information 2 Supporting data 2: A Tree map representation of keywords plus (top 30) based on frequency of usage. Each box represents a keyword, the number of usages of the keywords, and the frequency of usage of these keywords. [file CJID-2026-2362823-s002.docx]
